# A Melanin-Deficient Isolate of *Venturia inaequalis* Reveals Various Roles of Melanin in Pathogen Life Cycle and Fitness

**DOI:** 10.3390/jof9010035

**Published:** 2022-12-25

**Authors:** Ulrike Steiner, Erich-Christian Oerke

**Affiliations:** INRES–Plant Pathology, Rheinische Friedrich-Wilhelms-Universitaet Bonn, Nussallee 9, 53115 Bonn, Germany

**Keywords:** melanin mutant, pathogenicity, stress tolerance, fungicide sensitivity

## Abstract

*Venturia inaequalis* is the ascomycetous pathogen causing apple scabs and forms dark-pigmented spores and partially melanised infection structures. Although melanin is considered to be essential for the infection of host tissue, a spontaneously occurring melanin-deficient mutant was isolated from an abaxial side of an apple leaf and can be cultivated in vitro as well as in vivo. The morphology and development of the melanin-deficient-isolate SW01 on leaves of susceptible apple plants were compared to that of the corresponding wild-type isolate HS1. White conidia of SW01 were often wrinkled when dry and significantly increased their volume in suspension. Germination and formation of germtubes and appressoria were not impaired; however, the lack of melanisation of the appressorial ring structure at the interface with the plant cuticle significantly reduced the infection success of SW01. The colonisation of leaf tissue by non-melanised subcuticular hyphae was not affected until the initiation of conidiogenesis. Non-melanised conidiophores penetrated the plant cuticle from inside less successfully than the wild type, and the release of white conidia from less solid conidiophores above the cuticle was less frequent. Melanin in the outer cell wall of *V. inaequalis* was not required for the survival of conidia under ambient temperature or at −20 °C storage conditions, however, promoted the tolerance of the pathogen to copper and synthetic fungicides affecting the stability and function of the fungal cell wall, plasma membrane, respiration (QoIs) and enzyme secretion, but had no effect on the sensitivity to sulphur and SDHIs. The roles of melanin in different steps of the *V. inaequalis* life cycle and the epidemiology of apple scabs are discussed.

## 1. Introduction

*Venturia inaequalis* has been characterised as a dark-pigmented ascomycetous fungus with an electron-translucent inner layer and an electron-dense outer layer of the cell walls of ascospores and hyphae, which may resemble dark granules of melanin [1,2]. The two-celled ascospores are olive-brown, the conidiophores are pale to mid-brown to olivaceous-brown, and produce pale to mid-olivaceous brown conidia [3]. On apple leaves, the strongly melanised conidia (and ascospores) produce almost hyaline germtubes which, in general, form light apical appressoria. Light microscopic studies demonstrate that neither the sub-cuticular hyphae produced after penetration of the cuticle are pigmented [4]. More than 50 years ago, the mycelium of *V. inaequalis* grown in liquid media has been shown to excrete a dark-brown protein reported to contain melanin [5,6]. The saprophytic mycelium formed on dead apple leaves is darkly pigmented as the pseudothecia are [7,8]. The biology of the apple scab fungus and the interactions with its host plant have been summarised by MacHardy [9] and Bowen et al. [10]; however, the role of melanin in the pathogenesis and epidemiology of *V. inaequalis* is rather obscure. Pigmentation of the colourless appressoria is limited to a melanised appressorial ring structure (MARS) surrounding the appressorial pore but is essential for the pathogenicity of *V. inaequalis* on apple leaves [11]. Hering et al. [12] described that cuticle penetration may occur without the previous formation of an appressorium; this could limit the role of melanin as a prerequisite for pathogenicity in *V. inaequalis*.

Melanins are dark-brown to black pigments not essential for growth and development, but suitable to enhance the survival and competitive abilities of species in certain environments [13]. Various fungi synthesise melanin from tyrosine via 3,4-dihydroxyphenylalanine (DOPA) in culture media. Melanins in cell walls of the Basidiomycota are derived from γ-glutaminyl-3,4-dihydroxybenzene or catechol, as the immediate phenolic precursor of the melanin polymer. In the Ascomycota and related Deuteromycetes, the dark-brown to black melanins in cell walls are generally synthesised from the pentaketide pathway in which the polyketide synthase (PKS) is the key enzyme and 1,8-dihydroxynaphthalene (DHN) is the immediate precursor of the polymer [14]. *Venturia inaequalis* is described as forming melanin via the DHN pathway [11,15]. The extracellular dark pigments produced by fungi may be formed from various fungal phenols, other microbial phenols and plant phenols [13].

Melanin is located within the fungal cell wall, but distribution and quantity vary widely among species. *Cryptococcus neoformans*’ melanin is first detectable along the plasma membrane and fills throughout the cell wall over time. In contrast, melanin is located along the outer regions of the cell wall and/or clustered on the cell wall surface of several fungi pathogenic to humans, including *Aspergillus* spp. [16]. The composition of the outer cell wall of *Aspergillus* sp. varies between morphotypes, hyphae, and conidium which has a rodlet layer composed of hydrophobins followed by DHN melanin [17,18]. Electron microscopic techniques have indicated that the layers or clusters of melanin are formed by granules of the polymer [16].

In the plant pathogen *Colletotrichum graminicola*, melanin apposition occurs as a thin layer directly adjacent to the cell membrane but seems to affect also the surface of the appressorial cell wall [19]. In melanin-containing wild types (WT), the surface was more indented than in melanin-deficient mutants. Similarly, melanin is located adjacent to the plasma membrane of *Magnaporthe oryzae* appressoria [20,21]. In *Alternaria* spp., melanin is confined to the outer region of the primary conidial cell walls and the septa delimiting individual cells in the multicellular conidium [22,23]. Disruption of a melanin biosynthesis gene in *A. alternata* led to a reduction in the conidial size as well as the septal number [24]. *Alternaria* spp. produce non-melanised appressoria and melanin-deficient mutants of *A. alternata* and *A. brassicicola* retain their pathogenicity [25,26,27]. In *V. inaequalis*, melanin is deposited in the out layer of the cell wall of conidia, conidiophores and in the appressorial ring structure [11].

The complex chemical structure of fungal melanins lends them multiple unique functions ranging from radioprotection and antioxidant activity to heavy metal chelation and organic compound absorption [28]. Melanin is generally produced intracellularly and subsequently incorporated into the fungal cell wall for protection against ionising UV radiation and desiccation [29]. It can protect against antifungal drugs [30] and free oxygen radicals generated by host defence mechanisms [31,32]. DHN-melanin, among other tolerance mechanisms, significantly contributes to cadmium tolerance in more melanised dark septate endophytic fungi by immobilising Cd to hydroxyl groups and maintaining the integrity of the fungal cell wall [33]. A pksA knockout mutant of *Fonsecaea monophora* was more sensitive to oxidative stress, extreme pH environment, and antifungal drugs than the wild-type [34].

A correlation between pigments and resistance to ultraviolet radiation has been widely recognised in microorganisms; however, there is still some debate on this topic, with non-pigmented strains sometimes being more resistant than their pigmented counterparts [35]. Melanin-deficient conidia of three food-spoiling fungi were not altered in their heat resistance; however, had increased sensitivity towards UV-C radiation [36].

The isolation and maintenance of a melanin-deficient mutant of *V. inaequalis* spontaneously occurring on the adaxial leaf side of an apple plantlet in scab experiments under controlled conditions, enabled targeted experiments to elucidate the role of melanin in the development of the apple scab fungus. The melanin-deficient isolate called ‘SW01′ is considered to be a subculture of the wild-type isolate ‘HS1′. As the molecular background of SW01 is currently under investigation, here we focus on the occurrence–stage of pathogenesis and spatial pattern and biological role of melanin for *V. inaequalis*.

## 2. Materials and Methods

**Pathogen**. Conidia of *V. inaequalis* strains HS1 and SW01 were stored on infected apple leaves at −20 °C. Strain HS1 has been isolated from leaves of apple trees grown in Bonn-Poppelsdorf, Germany, in 1995; the melanin-deficient isolate SW01 was sub-cultured from apple leaves infected by HS1 and grown in a climate chamber in 2011. White conidia from an abaxial scab colony were propagated on susceptible leaves of apple cultivars ‘Golden Delicious’ and ‘Cripps Pink’. ITS sequencing primers ITS 1 and ITS 4–of isolate SW01 revealed 98 to 99% sequence identity with *V. inaequalis* strains CBS 180 47, CBS 330 65, and CBS 814 9. Conidia were washed off diseased leaves and were used for inoculation experiments at a conidia concentration of typically 5 × 10^4^ conidia ml^−1^. Other concentrations used are mentioned in the Results section.

**Plant cultivation**. Seeds of *Malus* x *domestica*, cv. ‘Cripps Pink’ stored at −18 °C were soaked in water for three days before incubation at 4–7 °C under high relative humidity (RH) conditions for 3 weeks. After sowing in the substrate for salt-sensitive plants (pH 5 to 6, salt content: 0.8 g L^−1^, Balster Einheitserdewerke GmbH, Froendenberg, Germany), they grew at 18 to 20 °C and 16 h daylight in a greenhouse. After 2 weeks, individual seedlings were grown in plastic pots (8.5 × 8.5 × 7.5 cm) with a standard potting mixture (Balster Einheitserdewerke GmbH), irrigated, and fertilised (liquid fertiliser Flory 2 special, 16-9-22) as required. Powdery mildew infection was avoided by the application of sublimate sulphur. Plants with a minimum of 6 leaves were used for the experiments.

**Growth on artificial media**. Freshly harvested conidia were collected in water and amended with antibiotics (penicillin, streptomycin, ampicillin, and chlortetracycline with a final concentration of 100 ppm each). Several droplets were placed on malt extract agar (48 g malt extract (Merck, Darmstadt, Germany), 5 g agar (Merck), 1 g yeast extract (Merck), 2 g D(+)-glucose (AppliChem GmbH, Darmstadt, Germany) per litre, pH 5.8) and potato dextrose agar (39 g potato dextrose agar L^−1^ (Merck) and incubated at 18 °C for two weeks at least.

**Inoculation and disease assessment**. Plants were spray-inoculated on the adaxial leaf side by using a hand-held sprayer. After incubation at 100% RH, 20/18 °C for 48 h, they were grown at 50 to 65% RH, 20/18 °C in climate chambers with artificial light for 16 h. Disease severity on apple leaves was assessed 7 to 16 days post-infection (d p.i.) as a percentage of leaves displaying typical leaf scab symptoms (=leaf area with brown and white conidia, respectively, n = 6 to 10).

**Fungicide application**. Plants were sprayed with fungicides 1 day prior to inoculation. The fungicides were applied at 100, 30, 10, 3, 1, 0.3, and 0.1% of the recommended dose. Compounds with one active ingredient only were used: boscalid (Cantus^®^, BASF SE, Limburgerhof, Germany); carpropamid (Bayer Crop Science, Monheim, Germany); copper hydroxide (Cuprozin Progress^®^, Spiess-Urania Chemicals, Hamburg, Germany); cyprodinil (Chorus^®^, Syngenta Agro GmbH, Frankfurt, Germany), Difenoconazole (Score^®^, Syngenta Agro GmbH), kresoxim-methyl (Discus^®^, BASF SE), sulphur (Kumulus S^®^, BASF SE). The non-formulated carpropamid was first solved in 1 mL acetone and diluted with water containing 0.03% emulsifier NP-15.

**Light microscopy**. Droplets (5 µL) of *V. inaequalis* conidia suspensions were incubated on glass slides at ambient temperature and 100% RH. Leaf discs were excised from the 2^nd^ to 3^rd^ youngest leaves of apple plants by using a cork borer (Ø 1.1 cm). They were cleared in saturated chloral hydrate (Sigma-Aldrich, Darmstadt, Germany) for 3 to 5 days.

For brightfield microscopy, specimens were stained with acid fuchsin (Merck, Darmstadt, Germany; 0.01 % in lactophenol) or aniline blue according to Bruzzesse and Hasan [37] for protein staining. For fluorescence microscopy of fungal structures, leaf samples were rinsed with distilled water and stained with Uvitex 2B (0.05%; Polysciences, Warrington, USA). The stained pathogen structures were visualised using filter cube A (excitation 340–380 nm, beam splitter 400 nm, stop filter LP 425 nm). For imaging of total preparations and conidia, samples were studied under a light microscope DM6000 B (Leica, Wetzlar, Germany) equipped with Nomarski interference contrast and epifluorescence, respectively. Images were recorded and analysed using the software Diskus (v. 4.60.1611; Technisches Buero Hilgers, Koenigswinter, Germany).

**Scanning electron microscopy** (SEM) of infected apple leaves was performed according to Juraschek et al. [38].

**Transmission electron microscopy** (TEM). The preparation and examination of samples were performed as described by Schumacher et al. [39].

## 3. Results

### 3.1. Phenotype of Venturia inaequalis Isolates on Apple Leaves and Artificial Medium

**Figure 1:**
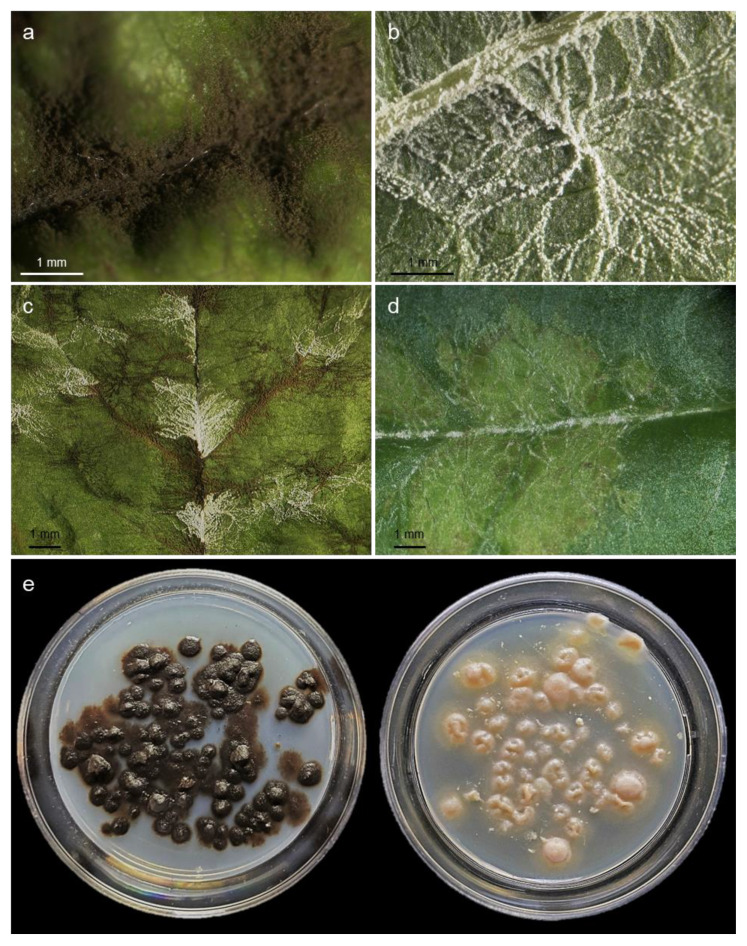
Symptoms of *Venturia inaequalis* isolate HS1 (wild-type; (**a**,**c**)) and SW01 (melanin-deficient mutant, (**b**–**d**)) on apple leaves. The wild-type produces large numbers of melanised conidia on the leaf surface, 10 dpi (**a**); the melanin-deficient isolate produces white conidia preferably on leaf veins, 10 dpi (**b**); sporulation of both isolates after mixed inoculation, 10 dpi (**c**); formation of conidia and chlorotic areas by SW01, 18 dpi (**d**). Pure cultures of HS1 (**left**) and SW01 (**right**) displaying the typical pigmentation of *V. inaequalis* mycelium on potato dextrose agar (**e**).

Leaf scabs caused by the wild-type HS1 and the melanin-deficient isolate SW01 differed in colour–brown and white, respectively–as well as in intensity (Figure 1). The wild-type isolate produced more conidia and intensively colonised not only the leaf veins but regularly also the intercostal leaf areas. The white conidia of SW01 were preferably produced on leaf veins of higher order whereas the colonisation of intercostal areas often resulted in the formation of chlorotic spots with poor sporulation (Figure 1d). Due to the white colour of conidia, the symptoms of SW01 were better noticeable on the green leaves than the dark brown scab symptoms of the wild type. The chlorotic spots produced by SW01 infections became visible at 14 to 15 dpi.

Both isolates could be cultured on artificial media such as potato dextrose agar or malt extract agar (Figure 1e). On agar, both isolates did not differ in growth rate and were able to produce conidia.

### 3.2. Morphological Characteristics of Conidia of Venturia inaequalis Isolates

**Figure 2:**
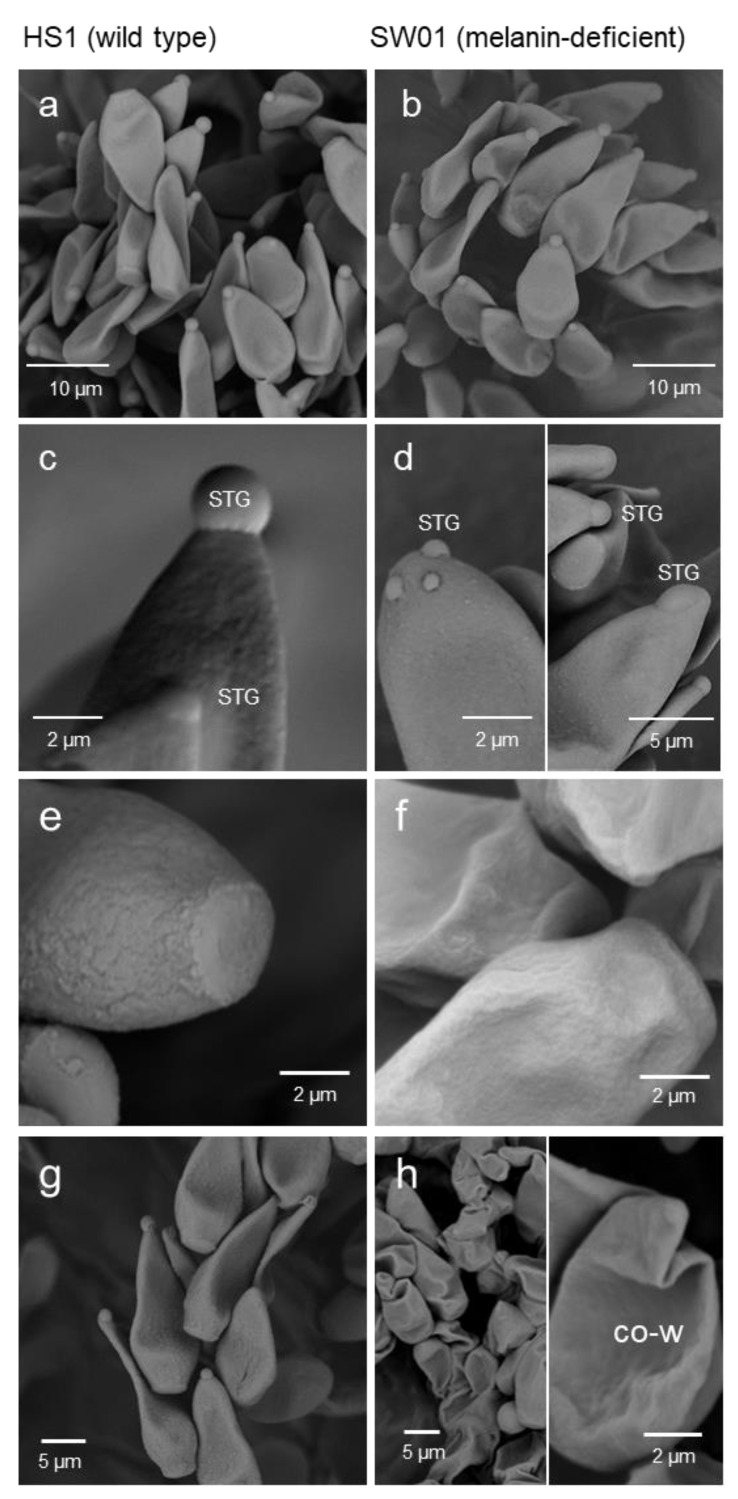
Scanning electron microscopic images of conidia of *Venturia inaequalis* isolates HS1 (wild-type; (**a**,**c**,**e**,**g**)) and SW01 (melanin-deficient, (**b**,**d**,**f**,**h**)). Mature conidia of both isolates showed an apical spore tip glue (STG; (**a**,**b**)), which was perfectly centred for isolate HS1 (**c**), and often disaggregated into small droplets or dislocated from the apex for SW01 ((**d**), **left**, **right**); the surface of melanised HS1 conidia showed incrustations, the basal part was almost perfectly roundish and rarely shrivelled (**e**), in contrast to SW01 with rather smooth surface and shrivelled conidia base (**f**); differences in the rigidity of the cell wall resulted in straight and strongly wrinkled dry conidia (co-w) of HS1 and SW01, respectively (**g**,**h**).

Conidia of the melanin-deficient isolate SW01 proved to be pure white and had a smoother surface than the wild-type isolate HS1 (Figure 2e,f). Dry conidia harvested from diseased leaves were significantly shorter resulting in a reduced length/width ratio (Table 1). After suspending in water, the length and width of hydrated SW01 conidia increased by 23% and 31%, respectively, and resulted in significantly wider conidia compared to HS1. The shape of the wild-type conidia was stable throughout hydration, whereas the increase in conidia size from hydration indicated a more flexible structure of the melanin-deficient conidia. The droplet of spore tip glue (STG) was positioned on the tip of wild-type conidia, whereas these droplets were less frequent on SW01 conidia and their position varied from the tip to more basal parts of conidia (20 to 30% of conidia; Figure 2a–d). Dehydration of SW01 conidia resulted in the shrinking and collapse of the pear-like shape, often the tip was wrinkled. In contrast, wild-type conidia remained straight and the loss in volume was lower (Figure 2g,h). Transmission electron microscopy confirmed the presence of melanin in the outer cell wall of HS1 conidia and the lack of melanin in the cell wall of isolate SW01 (Figure S1).

### 3.3. Pathogenesis of Venturia inaequalis Isolates

#### 3.3.1. Germination and Appressoria Formation

**Figure 3:**
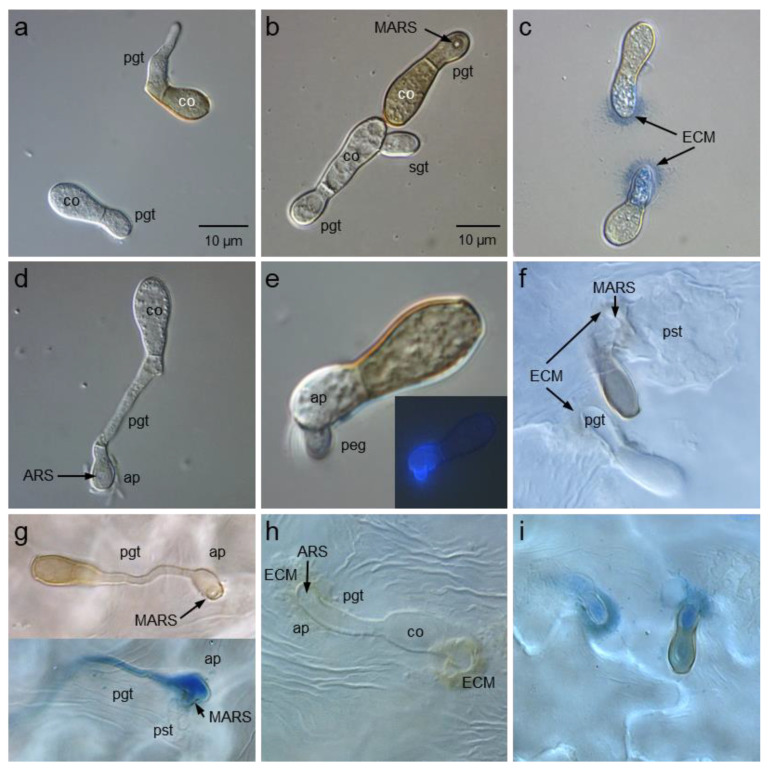
Germination of conidia of the *Venturia inaequalis* isolates HS1 (wild-type) and SW01 (melanin-deficient-mutant) on glass surfaces (**a**–**e**) and penetration of apple leaf surfaces after formation of appressoria (**f**–**i**). Melanised conidia (co) most often produced only a short primary germtube (pgt; (**a**,**b**)) and an appressorium with a melanised appressorial ring structure (MARS, (**b**)), whereas SW01 conidia often produced a second germtube (sgt; (**b**)). The formation of appressoria (ap) was associated with the release of an extracellular matrix (ECM) surrounding the appressoria of both isolates (**c**,**f**,**h**); The appressorial ring structure (ARS) of SW01 appressoria remained non-melanised (**d**); the wild-type isolate sometimes formed a penetration peg even on a glass surface; fluorescence images of aniline blue-stained samples (insert) confirmed the absence of melanin from the appressorium body and the peg (**e**), whereas the appressorial ring structure at the interface of appressorium and leaf cuticle was melanised and resulted in a primary stroma (ps) beneath the plant cuticle for the wild-type isolate HS1 (**g**); ECM around appressoria produced by isolate SW01 were often larger than ECM around HS1 appressoria (**h**,**i**).

Conidia germination, germtube growth and the formation of appressoria were investigated on glass slides as well as on apple leaf tissue. The germination of hydrated conidia started with the swelling of the conidial tip after 1 to 2 h for both isolates. The rate of germination on the artificial surface was reduced for SW01, however, the length of germtubes was not affected (Table 2). SW01 conidia often produced a secondary germtube independent of the underlying surface (Figure 3b, Table 3). The rate of appressoria formation per germtube and the formation of the (melanised) appressorial ring structure (M)ARS were significantly lower for the melanin-deficient isolate.

On leaves of the scab-susceptible apple cv. ‘Cripps Pink’, the germination rate of isolate SW01 was no reduced 96 h p.i. and germtubes had almost double the length of germtubes produced by HS1 conidia. The reduction in the rate of appressoria formation was less pronounced than on glass slides, however, formation of the non-melanised appressorial ring structure at the interface between appressorium and leaf cuticle was drastically reduced. This reduction resulted in differences in the successful formation of the sub-cuticular primary stroma which was reduced by 90% for SW01 as compared to the wild type. Appressoria of both isolates were often surrounded by extracellular material stainable with aniline blue (Figure 3c,g,i); the formation of this extracellular matrix was significantly higher for the melanin-deficient isolate (Table 3). The leaf cuticle was penetrated by a non-melanised peg in both isolates and resulted in the formation of a primary stroma which was non-melanised for both isolates as well (Figure 3f).

**Table 1:** Morphology of dry and hydrated conidia of *Venturia inaequalis* isolates HS1 (wild type) and SW01 (melanin-deficient). Mean ± standard error of the mean (n = 200).

	HS1	SW01	Statistics ^1^
Dry conidia:			
Length [µm]	18.21 ± 0.15	16.46 ± 0.16	*p* ≤ 0.001
Width [µm]	7.81 ± 0.07	7.90 ± 0.08	*p* = 0.731
Ratio length/width	2.36	2.13	*p* ≤ 0.001
Conidia in water:			
Length [µm]	20.62 ± 0.18	20.31 ± 0.15	*p* = 0.316
Width [µm]	8.86 ± 0.07	10.31 ± 0.07	*p* ≤ 0.001
Ratio length/width	2.35	2.06	*p* ≤ 0.001
Ratio dry: wet	*p* = 0.759	*p* = 0.176	
Increase in water	L 13.2%/W 13.4%	L 23.4%/W 30.5%	

^1^ Mann–Whitney U test.

**Table 2:** Development of *Venturia inaequalis* isolates HS1 (wild type) and SW01 (melanin-deficient) on glass slides at 22 ± 2 °C, 16 h p.i. Mean ± standard error of the mean (n = 700).

Isolate	Germination Rate [%]	Germtube Length [µm]	Appressoria Per Germtube	(M)ARS Per Germtube
HS1	71.5 ± 2.2	17.7 ± 0.5	0.37 ± 0.03	0.16 ± 0.02
SW01	54.2 ± 1.7	16.1 ± 0.6	0.20 ± 0.03	0.07 ± 0.01

**Table 3:** Development of *Venturia inaequalis* isolates HS1 (wild type) and SW01 (melanin-deficient) on apple leaves, 96 h p.i. Mean ± standard error of the mean (n = 307).

	HS1	SW01	Statistics ^1^
Germination rate [%]	88.4	90.8	*p* = 0.020
Multiple germination [%]	2.8	29.6	*p* ≤ 0.001
Germtube length [µm]	20.0	35.5	*p* ≤ 0.001
Appressoria per conidium	0.726	0.532	*p* ≤ 0.001
(M)ARS per conidium	0.503	0.070	*p* ≤ 0.001
Appressorium with extracellular matrix [%]	7.6	38.0	*p* ≤ 0.001
Subcuticular infection structure (stroma) [%]	29.6	2.8	*p* ≤ 0.001

^1^ Mann–Whitney U test.

#### 3.3.2. Subcuticular Growth

**Figure 4:**
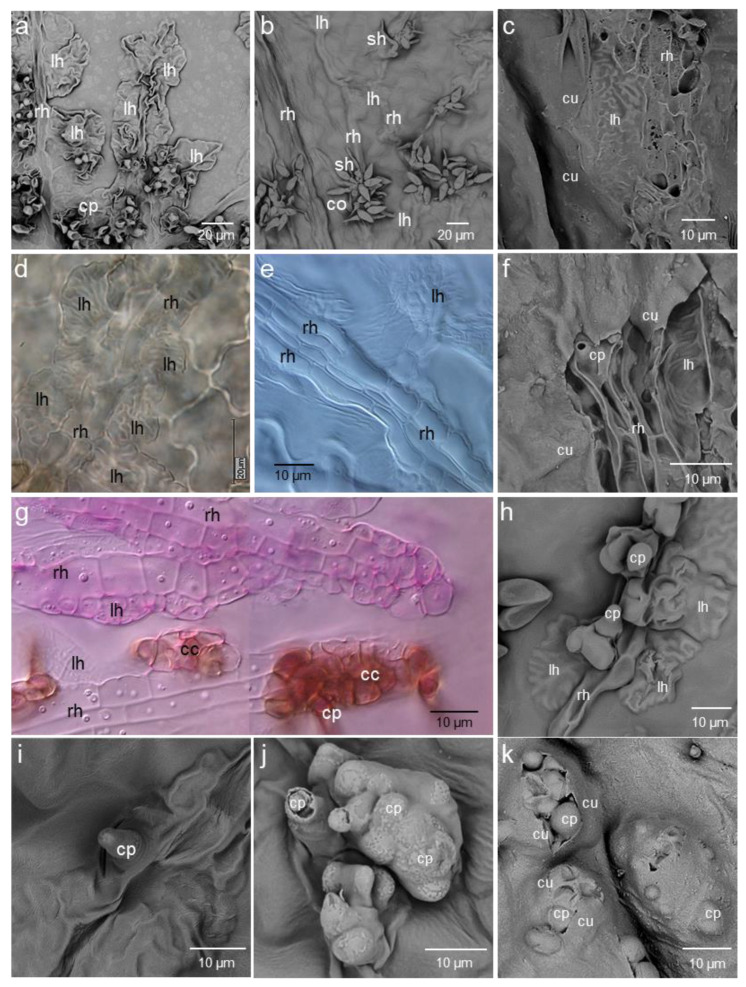
Sub-cuticular development of Venturia inaequalis isolates HS1 (wild-type, (**a**,**d**,**g**,**h**,**j**)) and SW01 (melanin-deficient-mutant, (**b**,**c**,**e**,**f**,**g**,**i**,**k**)). Overview on structures visible on the surface of apple leaves with runner hyphae (rh), lobated hyphae (lh), and the formation of conidiophores (cp) and conidia (co) breaking through the plant cuticle (**a**,**b**). Fungal structures on the epidermal cells of leaf tissue after cuticle removal (**c**,**f**); formation of runner hyphae and lateral lobated hyphae of *V. inaequalis* isolates HS1 (**d**) and SW01 (**e**); sub-cuticular hyphae of isolate SW01 ((**g**), **top**) and HS1 ((**g**), **bottom**) in the same leaf tissue with melanised conidiogenic cells (cc) for HS1 only; formation of conidiophores of HS1 (**h**) and SW01 (**i**) still covered by the leaf cuticle; penetration of the cuticle by the pathogen’s structure, formation of holes in the cuticle (**j**,**k**). Images of samples taken 8 to 10 dpi from scanning electron microscopy (**a**–**c**,**f**,**h**–**k**) and brightfield microscopy (**d**,**e**,**g**), without and with staining (staining with acid fuchsin in (**g**)).

Subcuticular growth of both *V. inaequalis* isolates was very similar and finally resulted in sporulation (Figure 4). Starting from the primary stroma, *V. inaequalis* formed non-melanised runner (stolon-like) hyphae—often two in parallel—which produced lateral lobate hyphal structures. These very thin and laminar fan-shaped hyphae were highly organised by cell wall indentions detectable in light and electron microscopic images. The runner hyphae, in contrast, were filamentous and almost round in diameter (Figure 4e,f). Brightfield microscopy revealed the formation of dot-like structures at the interface of the lower surface of the subcuticular fungal cell wall and the epidermal cell wall beneath for both isolates (Figure 4g). When reaching a critical sub-cuticular biomass, *V. inaequalis* produced a secondary stroma comprising more compact cells on top of fan-shaped and runner hyphae. At this stage of pathogenesis, the production of melanin of isolate HS1 resulted in the brownish melanisation of these cells and of conidiophore primordia still covered by the plant cuticle (Figure 4h,i). The secondary stroma increased in size and bulged the cuticle which was penetrated subsequently by the conidiophore often bearing vesicle-like primordia of conidial cells. After penetration, the plant cuticle tightly surrounded the base of the conidiophore and largely reduced the damage to the transpiration barrier to a minimum.

#### 3.3.3. Formation of Conidia

**Figure 5:**
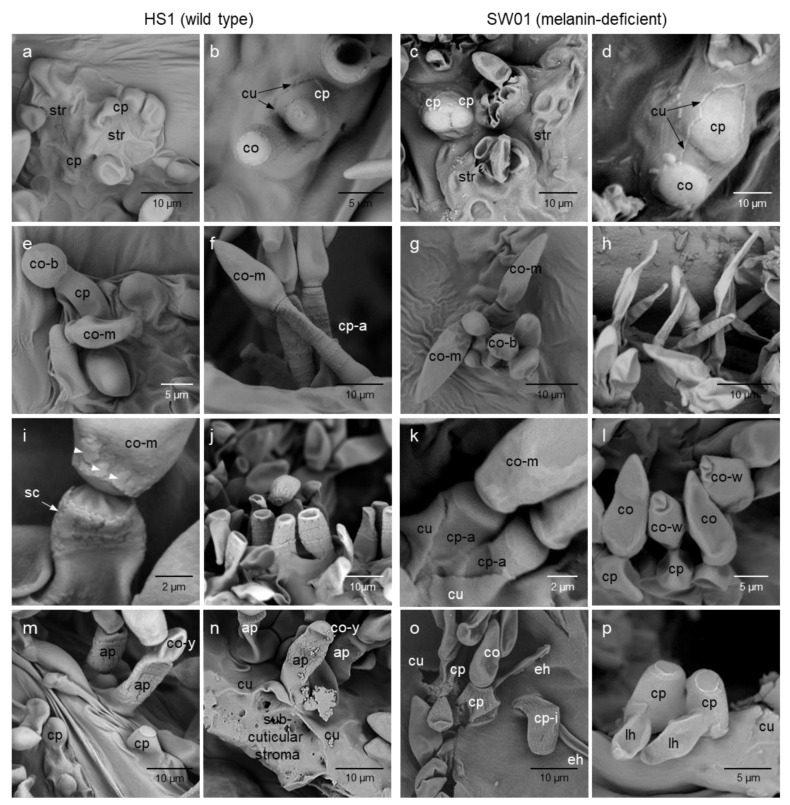
Conidiogenesis of *Venturia inaequalis* isolates HS1 (wild-type, (**a**,**b**,**e**,**f**,**i**,**j**,**m**,**n**)) and SW01 (melanin-deficient mutant; (**c**,**d**,**g**,**h**,**k**,**l**,**o**,**p**)) on apple leaves. Formation of secondary stroma (str) with conidiophores (cp) before (**a**,**c**) and after penetration of the plant cuticle (**b**,**d**); the cuticle tightly encloses the conidiophore, start of conidia (co) formation; conidia formation begins with an epicuticular balloon-like structure (co-b) which matures into the typical pear shape of mature *V. inaequalis* conidia (co-m; (**e**,**g**)); older conidiophores become typically annellate for HS1 (cp-a; (**f**)), whereas conidiophores of SW01 often are transformed into superficial hyphae (**h**); release of mature conidia (co-m) from conidiophores for HS1 and SW01 with and without prominent scars (sc) on the surface of conidiophore and conidium, respectively (**i**,**k**); remaining annellate conidiophores of HS1 (**j**) and wrinkled conidia (co-w) of SW01 still attached to the conidiophores (**l**); formation of a new conidium (co-y) on an older conidiophore (**m**), link between sub-cuticular stroma and epicuticular conidiophore (N) for HS1; conidiophores of SW01 sometimes lack rigidity (cp-i; (**o**)) or form lateral hyphae (lh) resulting in epicuticular hyphal growth (**p**). Scanning electron images taken at different stages of sporulation (6 to 14 dpi).

Small, balloon-like non-melanised conidia matured into typical pear-shaped, melanised conidia within some hours for *V. inaequalis* isolate HS1 (Figure 5a,b,e,f). The release of mature conidia was associated with the formation of scars on the tip of the conidiophore and the bottom of the conidium (Figure 5i) which was largely dehydrated in in vivo images (not shown), The successive formation of conidia resulted in annellide structures of the cell wall on the top of conidiophores (Figure 5j). For the melanin-deficient mutant, conidia formation was similar in the first stages (Figure 5c,d,g); however, the detachment of mature conidia could be observed much less frequently.

Conidia were often wrinkled and frequently remained on the conidiophore; although successive generation of conidia on a conidiophore was possible, including the formation of less pronounced scars and annellide structures on the conidiophore cell wall–the conidiophores were less stable, were bended and produced superficial hyphae at their basis instead of conidia on their top (Figure 5l,o,p). Brightfield microscopy highlighted the differences in the pigmentation of conidiophores and conidia between *V. inaequalis* isolates; the melanised conidiophores of the wild-type isolate HS1 produced hyaline early stages of conidia which could be stained with acid fuchsin as well as with the combination of acid fuchsin and aniline blue (Figure S2). The hyaline conidiophores of isolate SW01 produced conidia that remained hyaline (and stainable) throughout their life span.

**Figure 6:**
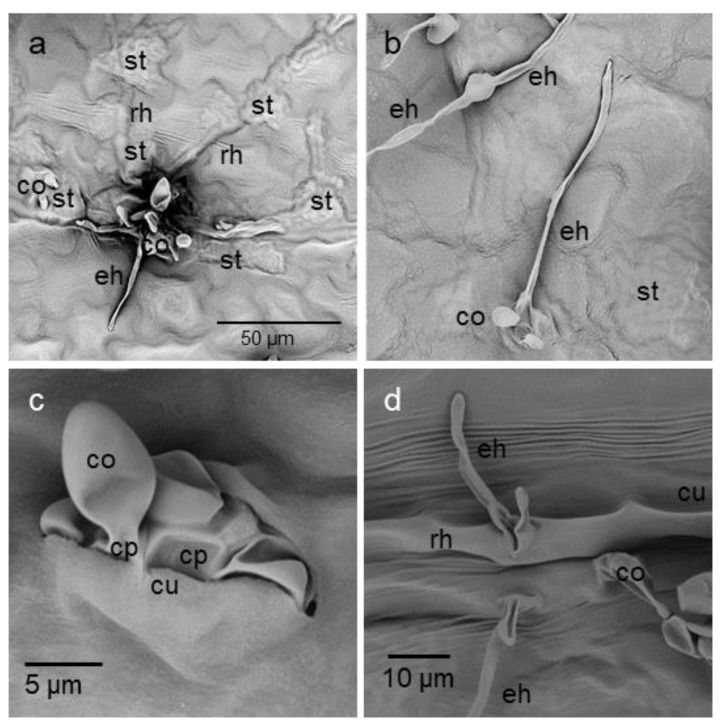
Pathogen structures on chlorotic tissue of an apple leaf 19 dpi with *Venturia inaequalis* isolate SW01. Rare and low formation of conidia (co) despite of strong sub-cuticular formation of runner hyphae (rh) and secondary stroma (st) with lobated hyphae (**a**); formation of lateral epicuticular hyphae (eh) from conidiophores (**b**); penetration of the cuticle by conidiophores with young conidia (**c**); formation of epicuticular hyphae directly from sub-cuticular runner hyphae, formation of a conidium from neighbouring stroma (**d**). Scanning electron microscopic images.

For latent infections associated with the formation of chlorotic spots, the sub-cuticular colonisation of leaf tissue by SW01 was intense, but the formation of conidia was usually poor. Instead, narrow epicuticular hyphae were formed on the base of conidiophores and even from runner hyphae (Figure 6).

### 3.4. Aggressiveness of the Melanin-Deficient Mutant on Apple Leaves

When inoculated with the same inoculum density, the reduced ability of isolate SW01 to infect and colonise apple leaves resulted in a significantly lower percentage of diseased leaf area as compared to the wild-type isolate HS1 (Table 4). This proved to be true for various inoculum densities. The lower aggressiveness of SW01 became even more obvious when the isolates were inoculated in the mixture. Despite the higher percentage of SW01 in the mixture (60:40), the percentage of the resulting brown scab was 25 to almost 50 times higher than that of the whitish scab.

**Figure 7:**
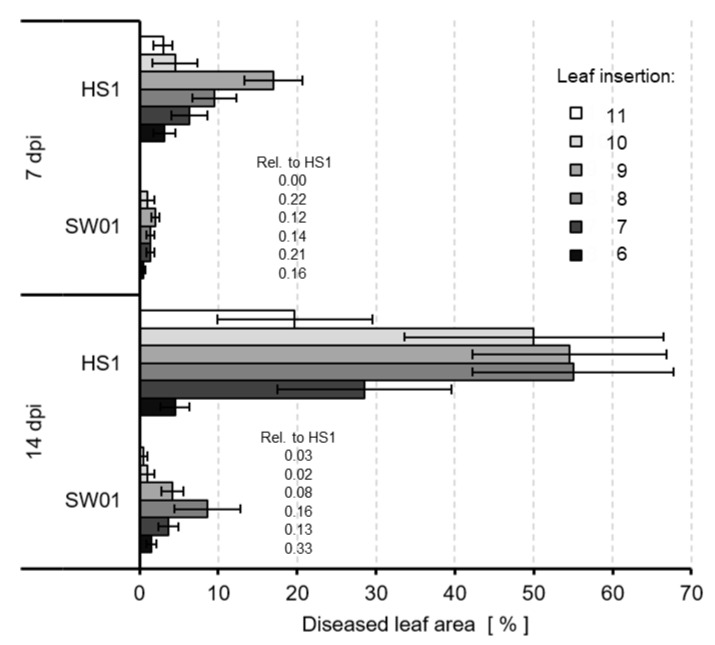
Effect of leaf age on the severity of leaf scab caused by *V. inaequalis* isolates HS1 (wild type) and SW01 (melanin-deficient mutant) 7 and 14 d p.i., respectively. Leaf insertion was counted from bottom to top. Bars indicate standard error of the mean (n = 6). The relative portion of SW01 on older leaves was higher than on the more susceptible, younger leaves.

The effect of plant resistance depending on leaf age on infection success was similar but not identical for both isolates of *V. inaequalis* (Figure 7). The intensity of the scab decreased from the youngest leaf unfolded at the time of inoculation to older leaves, as measured as a percentage of diseased leaf area. Surprisingly, the disease intensity of the less aggressive isolate SW01 was higher on the more resistant older leaves in relative terms; in some experiments, the melanin-deficient isolate produced white scab lesions on a leaf level the brown scab showed no symptoms.

Experiments with *V. inaequalis* inoculation and incubation for 48 h under controlled conditions and subsequent incubation under artificial irradiation in a growth chamber or under full sunlight outdoors gave no evidence for a contribution of fungal melanisation in UV tolerance of the pathogen. The ratio between brown and white scab symptoms was independent of the incidence of UV light.

**Table 4:** Infectivity of *Venturia inaequalis* isolates HS1 (wild type) and SW01 (melanin-deficient) on apple leaves under greenhouse conditions, 12 d p.i. Mean ± standard error of the mean (n = 6).

Isolate	Diseased Leaf Area [%]			
	Pure Inoculum		Mixture (60:40, SWo1:HS1)	
	2.5 × 10^4^ co. mL^−1^	1 × 10^5^ con. mL^−1^	5 × 10^4^ con. mL^−1^	1 × 10^5^ con. mL^−1^
HS1 (wild type)	8.9 ± 1.1	19.8 ± 2.0	26.5 ± 4.6	27.4 ± 7.4
SW01	4.0 ± 0.7	8.3 ± 1.3	0.56 ± 0.24	1.1 ± 0.46
HS1:SW01	2.23:1	2.38:1	47.3:1	24.9:1

### 3.5. Sensitivity of V. inaequalis Isolates to Fungicides

The reaction of the wild-type and the melanin-deficient isolates to inorganic and synthetic fungicides with different modes of action on the fungal metabolism was assessed in inoculation experiments with a protective application (1 d before inoculation) of commercial fungicides with one active ingredient only. The EC_50_ values calculated from dose–response curves demonstrated significant differences in the susceptibility of isolates to fungicides (Table 5). The melanised wild-type isolate was highly susceptible to the melanin biosynthesis inhibitor carpropamid–in contrast to the melanin-deficient isolate SW01. The sensitivity of this isolate to inorganic sulphur and the carboxamide boscalid was similar to that of the wild type. It was, however, significantly more sensitive to copper hydroxide and synthetic compounds interfering with the secretion of hydrolytic enzymes (aniline-pyrimidine), ergosterol biosynthesis (triazole) and respiration complex III (quinone outside inhibitor) of fungi.

## 4. Discussion

Melanisation of *Venturia inaequalis* structures is limited to mature conidia and ascospores, conidiophores, a melanised ring structure of appressoria facing the surface of the host plant, and saprophytic hyphae produced after leaf fall; in contrast, germtubes, appressoria and the parasitic sub-cuticular structures–runner hyphae and fan-shaped hyphae covering the cell wall of epidermal cells–are not melanised. The loss of the ability to produce melanin in the melanin-deficient mutant SW01 was associated with a loss in cell wall rigidity, reduced aggressiveness to the host plant and higher vulnerability to some xenobiotics, but neither with a complete loss in pathogenicity nor a total loss in fitness and survival.

Complementation assays with a melanin-deficient mutant and treatments of wild-type isolates with MBIs demonstrate that *V. inaequalis* produces DHN melanin [11,15]. Growth of an albino mutant of *V. inaequalis* isolated from an orchard in Ontario, Canada, on a scytalone-amended medium resulted in the formation of dark granules similar to those seen in wild-type isolates [15]. The role of the shape of fungal spores and its stability for their function in dispersal and as an inoculum source is hardly known. The germination rate of non-melanised *V. inaequalis* conidia was hardly affected under optimal conditions, nevertheless, a loss in melanin formation reduced the physical stability of SW01 conidia and, even more important, the release of conidia from conidiophores. The initials of wild-type conidia, hyaline balloon-like structures produced on the pigmented conidiophores, develop into the typical pear-shaped and olivaceous conidia only in later stages of conidiogenesis (Steiner & Oerke, unpublished). The non-melanised mutant produced a lower number of conidia per conidiophores (as visible from the lower formation of annelids) and the release of conidia from the producing structure was impaired. The central connection between conidiophore and conidium often remained intact, suggesting that melanisation of fungal structures may be involved also in the programmed detachment of spores. The impairment of the formation of conidia per conidiophore promoted the formation of epicuticular hyphae produced on the basis of conidiophores in SW01. This type of epicuticular hyphae has been also described for melanised isolates [40], however, its frequency seems to be considerably higher in the melanin-deficient mutant. The position of the spore tip glue which is responsible for the adhesion of conidia on the wet apple surface was also negatively affected by the loss of melanin in the outer part of the conidia cell wall. Localised as a distinct droplet on the apex of pear-shaped conidia, the combination of glue and pear-shape conidia result in immediate contact of the conidial tip with and the maximum amount of glue material on the plant surface and optimal adhesion on the wet hydrophobic cuticle (form and function). In contrast, the more globose shape and often irregular distribution of lower amounts of glue material make the adhesion of SW01 conidia less effective and less reliable. Melanin may form a corset-like net within the cell wall and seems to be crucial for the optimal shape of conidia–drop-shaped with a distinct tip, important for adhesion on wet host surface and rain fastness.

In SEM images, the surface of SW01 conidia proved to be smoother than that of the rather rough surface of wild-type conidia, likely because of the lack of melanin granules in the outer layer of the cell wall (Figure S1). Inhibition of DHN-melanin synthesis by pyroquilon enhanced morphological changes (increased hyphal balloon size), characterised by thinner and less organised *Aspergillus infectoria* cell walls [41].

Perception of the anticlinal cell walls as preferred (=optimal) sites for appressoria formation was not affected in the melanin-deficient isolate. Adhesion to the plant cuticle and especially sealing of the interface between pathogen and plant were negatively affected, as the percentage of appressoria with surrounding matrix material and the total amount of this material significantly increased. The increased attraction of bacteria propagating around the appressoria, especially for isolate SW01, indicates an increased leakiness of fungal structures and/or an increased secretion of organic molecules like cell wall degrading enzymes. Melanoproteins produced by *V. inaequalis* were speculated to tether secreted CWDEs and facilitate a slow release of them [31].

Reduced aggressiveness (independent of the genotype and the predisposition of host tissue) resulted from a significantly increased failure of the appressorial penetration peg. The melanised appressorial ring structure is likely to play a key role in the enzymatic lysis of the plant cuticle. Physical force of the penetration peg is not suitable to penetrate the thin cuticle on top of the epidermal cell layer. Cutinase and other hydrolytic enzymes secreted by *V. inaequalis* appressoria have to be focused and concentrated at the interface between the pathogen structure and plant surface in order to enable an efficient penetration of the cuticle. Melanin effectively focuses cutinase activity below the appressorial pore and, therefore, guarantees the penetration success of the pathogen. The total amount of enzymes per appressorium is sufficient to locally macerate the cuticle and allow the growth of the penetration hypha through it and later between the cuticle and cell wall. The higher rate of appressoria surrounded by matrix material in the melanin-deficient isolate supports the idea of enzyme leakage which could explain the infection success on more resistant leaf insertions. The ring structure itself is likely to reduce the dispersal of enzymes even without melanin incrustation and allows a low rate of cuticle penetration. As the cuticle above the primary (and secondary) stroma remains functional, hydrolysis of the interface between the cuticle and cell wall should not include cutinases during subcuticular growth. Endo- and exo-polygalacturonase-like activities have been reported during in vitro growth of the pathogen [42,43].

The secretion of exoenzymes necessary for successful infection and colonisation of the host tissue should not be inhibited by melanin. However, the melanin content of *Cochliobolus sativus* mycelium was negatively correlated to extracellular enzymes and the aggressiveness of isolates [44]. Whether the melanin-deficient mutant secretes more hydrolytic enzymes has to be proven. The increased infection success on age-dependent resistant apple leaves may be attributed to a higher total cutinase activity of SW01 appressoria which is not focused on the tiny space within the appressorial ring structure. Experiments on appressoria formation on artificial surfaces at lower temperatures demonstrated that *V. inaequalis* intensifies the thickness of the (melanised) appressorial ring as long as the penetration attempt is not successful and the energy supply is sufficient (Steiner and Oerke, unpublished).

Especially infection and sporulation (=penetration of the cuticle from outside and from inside, respectively) were reduced in the melanin-deficient isolate, whereas the sub-cuticular development was not affected as these hyphae are not melanised. Melanin may be not essential for the pathogenicity of *V. inaequalis*, but it drastically increases the success of infection and sporulation, thus the epidemic spread of the disease. The significance of melanin in fungal pathogenicity may be similar to that in the protection of fungal hyphae against abiotic stress factors—not absolutely necessary but highly beneficial for the pathogen. Silencing of trihydroxynaphthalene reductase in *V. inaequalis* resulted in a distinctive light brown phenotype; however, maintained the ability to infect apple [45]. Maybe the mutant strain still produced melanin sufficient enough for successful pathogenesis [7].

The formation of melanin is under ontogenetic regulation; fungal structures permanently exposed to varying environmental conditions–ascospores, conidia and conidiophores, saprophytic hyphae and pseudothecia of the sexual stage–are melanised, whereas the subcuticular hyphae as well as germtubes and appressoria are non-melanised, except for the melanised appressorial ring structure on the host cuticle. Images of the growth of *V. nashicola* in Asian pear published by Park et al. [46] demonstrate that the subcuticular hyphae are not pigmented, whereas also this pathogen produces a melanised appressorial ring structure. In contrast, conidia of *V. carpophila* unsuccessful in infecting peach leaves produce melanised epicuticular runner hyphae [47]. The differential expression of melanin biosynthesis genes not only indicates to the economic efficiency of biosynthesis pathways –active only when needed–but also to the non-involvement of melanin in the nutrient uptake of *V. inaequalis*. A role of melanin and melanoproteins, produced in in vitro experiments in artificial liquid media by *V. inaequalis* [5,6] has been generalised to play a pivotal role also in parasitic nutrient uptake [9].

The formation of conidiophores, the transition from sheltered subcuticular growth to dispersal outside of the plant, is characterised by the switch to melanised cell walls. Interestingly the formation of conidia was significantly lower in the melanin-deficient isolate, most likely because of the low efficiency of the subcuticular structures to penetrate the cuticle from inside to the outside of the plant. Either the conidiophore or the emerging conidium has to penetrate the intact cuticle again in order to be able to release conidia above the cuticle. As the second cuticle penetration of *V. inaequalis* from inside to the outside was reduced similarly to the impaired penetration of the cuticle from outside to the subcuticular growth SW01 more often produced chlorotic spots with intensive subcuticular development of hyphae and nutrient uptake from the plant, however, only sparse sporulation. Although this phenomenon could be also detected for the wild type, it was much more frequent for the melanin-deficient isolate.

In *Stemphylium eturmiunum*, enhanced melanin biosynthesis correlates with an increased formation of propagules [48]. Conidiogenesis in black, white and mixed subpopulations of *Cochliobolus sativus* was positively correlated with the melanin content of mycelium [49]. In *M. oryzae*, the three melanin biosynthesis genes ALB1, RSY1 and BUF1 were required for conidiation in isolates Guy11 and 70-15, respectively. ALB1, RSY1, and BUF1 also conferred conidial resistance to environmental stresses (UV exposure, oxidisation, and freezing damage) [50].

The formation of non-melanised cells under the plant cuticle was not affected, but the success of breaching the cuticle by the conidiophore and/or the emergent conidia was significantly reduced. The combination of lower mechanical stability of conidiophores/conidia and correct positioning of enzymes to lyse the cuticle is a reasonable explanation. This failure results in extended endophytic growth with the formation of conidiophores and conidia under the cuticle, but the poor formation of epicuticular conidia–self-trapping below the cuticle because of the incapability to penetrate the cuticle.

For effective protection of fungal survival structures against microbial degradation and lysis and the impact of abiotic stress factors, melanin should be located in the outer cell wall layer; in contrast, for structures with a transient function in the fungal life cycle, e.g., generation of pressure for cell wall penetration, localisation in the inner cell wall layer is more appropriate.

Tolerance to abiotic factors such as drought, UV radiation, heavy metals and xenobiotica is relevant during periods of survival (sexual state) and spread (conidia). The hyphae and spores produced by *V. inaequalis* for these purposes/during these stages are melanised. In *Bipolaris oryzae* and *Botrytis cinerea*, light induces the activation of melanin biosynthesis genes [51,52]. Melanin is not likely to be necessary for protection against UV light or freezing–the storability of SW01 conidia at −20 °C was not affected when compared to HS1 (data not shown)–but for protection against microbial attack and toxic activity of xenobiotica. Melanin may confer resistance to hydrolytic enzymes produced by bacteria and other fungi, especially important for sclerotia-forming pathogens, those causing fruit mummies and surviving in the soil via spores [53,54,55]. Melanin expression in a transformant of *Metarhizium anisopliae* notably enhanced anti-stress capacity and virulence, in terms of germination, survival rate, and infectivity of diamondback moth compared with the wild type [56]. The shelf life of *V. inaequalis* conidia at −20 °C (dry, in suspension or on leaves) and at room temperature (dry, on leaves) was not affected for the melanin-deficient mutant. They remained infectious even after a storage period of more than six (room temperature) and twelve months, respectively.

Melanisation reduced the toxicity/uptake of copper and organic fungicides, especially for substances affecting the stability of fungal cell walls (such as MBIs) and plasma membrane (EBIs) and fungal respiration (QoIs), but had no effect on the sensitivity of *V. inaequalis* isolates to fungicides such as sulphur and SDHIs. A melanoprotein produced by *V. inaequalis* in liquid culture could be localised in the apoplast fluid of infected apple leaves. The melanoprotein binds to iron and copper [57] and may explain the protective activity against copper in HS1. The mode of action of anilinopyrimidines is reported to include the inhibition of methionine biosynthesis and the secretion of hydrolytic enzymes [58]. The role of cutinase release and its function in the penetration of the cuticle is likely to explain the higher sensitivity of the melanin-deficient isolate. *Alternaria infectoria* increased melanin deposition in cell walls in response to nikkomycin Z, caspofungin, and itraconazole, but not in response to fluconazole or amphotericin B [41]. Melanin is a significant contributor towards *Aspergillus brasiliensis* tolerance to biocides and photosensitisers [59]. This may be also playing a role in the higher competitiveness of HS1 in mixed inoculation with the melanin-deficient isolate lacking this protective activity.

## 5. Conclusions

The formation of melanin is not a prerequisite for the pathogenicity of *V. inaequalis* on apples; however, it dramatically increases the success of cuticle penetration and conidia release, contributes to the rigidity of cell walls and shape of conidia, and influences fungal tolerance to fungicides–it is, therefore, an important virulence factor of the apple scab pathogen. It is not absolutely necessary but promotes the fitness and survival of epiphytic fungal structures. Melanin does not contribute to nutrient uptake of *V. inaequalis* in vivo, as subcuticular runner hyphae and fan-shaped hyphae are not melanised. Melanin is indispensable for *V. inaequalis* to be a successful pathogen; nevertheless, its formation is restricted to structures and growth stages permanently exposed to the environment, whereas melanin-free structures within the plant are protected by the cuticle.

## Figures and Tables

**Table 5:** Sensitivity of the melanin-deficient-isolate SW01 of *Venturia inaequalis* to fungicides differing in the mode of action as compared to the wild-type isolate HS1. Evaluation of leaf scab (average of leaf levels 1 to 7), 12 d p.i. (n = 4). Mean disease intensity of non-treated plants was 20.7 % and 13.2 % for HS1 and SW01, respectively.

Fungicide		EC_50_ Value [mg L^−1^]		HS1 Rel.
Ingredient	Group	HS1	SW01	to SW01
Boscalid	SDHI ^1^ (C2)	5.6 ± 4.9	4.5 ± 1.9	1.23
Carpropamid	MBI ^2^ (I2)	5.1 ± 1.7	70 ± 67	0.07
Cyprodinil	AP ^3^ (D1)	3.2 ± 2.6	0.41 ± 0.79	7.80
Difenoconazole	Triazole (G1)	0.14 ± 0.53	<0.01	>14.0
Kresoxim-methyl	QoI ^4^ (C3)	0.18 ± 0.54	0.01 ± 0.02	14.28
Copper hydroxide	Inorganic (M)	>383	18 ± 12	>21.3
Sulphur	Inorganic (M)	114 ± 71	88 ± 25	1.30

^1^ Succinate-dehydrogenase inhibitors; ^2^ Melanin biosynthesis inhibitors; ^3^ Anilinopyrimidines; ^4^ Quinone outside inhibitors; FRAC code in brackets.

## Data Availability

Data supporting the reported results are available from the corresponding author, Erich-Christian Oerke, upon reasonable request.

## Supplementary Materials

The following supporting information can be downloaded at: https://www.mdpi.com/article/10.3390/jof9010035/s1, Figure S1. Melanisation of the outer cell wall of *Venturia inaequalis* conidia of the wild-type isolate HS1 and lack of melanin in the cell wall of the melanin-deficient isolate SW01. Figure S2: Staining of conidiophores and conidia of *Venturia inaequalis* as affected by the pigmentation of the wild-type HS1 and the melanin-deficient mutant SW01, respectively.
