# Supplementary material for: A Melanin-Deficient Isolate of Venturia inaequalis Reveals Various Roles of Melanin in Pathogen Life Cycle and Fitness
Source: J Fungi (Basel). 2022 Dec 25;9(1):35. doi: 10.3390/jof9010035 (PMC9867426; doi:10.3390/jof9010035)
Supplement: Supplementary file 1 [file jof-09-00035-s001.zip › jof-2101344-supplementary.pdf]

A Melanin-Deficient Isolate of *Venturia inaequalis* Reveals Various Roles of Melanin in Pathogen Life Cycle and Fitness

Ulrike Steiner and Erich-Christian Oerke

Rheinische Friedrich-Wilhelms-Universitaet Bonn, INRES – Plant Pathology,

Nussallee 9, 53115 Bonn, Germany

Supplementary Material

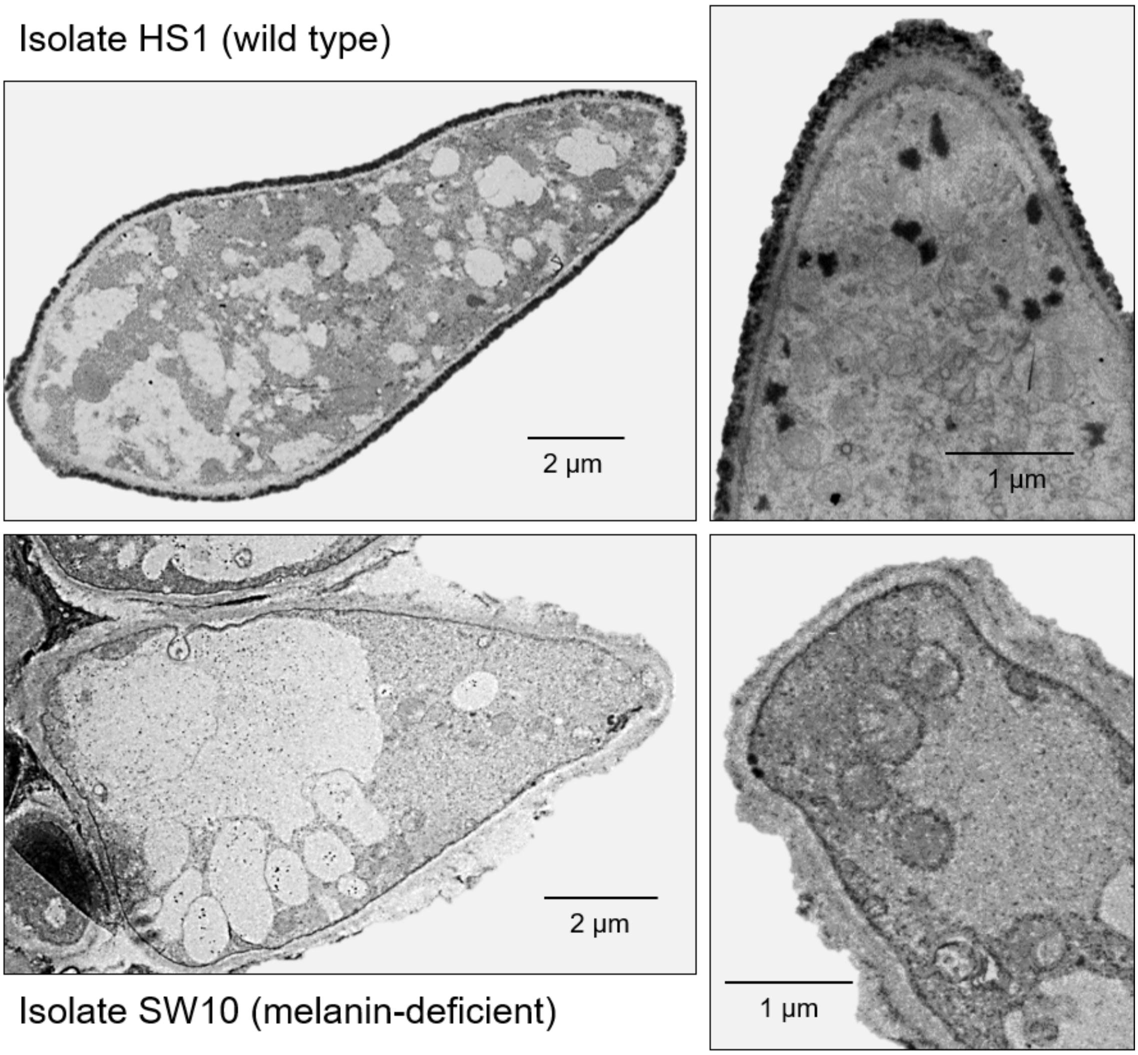

Figure S1. Melanization of the outer cell wall of *Venturia inaequalis* conidia of the wild-type isolate HS1 (top row; conidia [left], conidial tip [right]). Lack of melanin in the cell wall of the melanin-deficient isolate SW01 (bottom row; conidia [left], tip of conidiophore [right]). Transmission electron microscopy images.

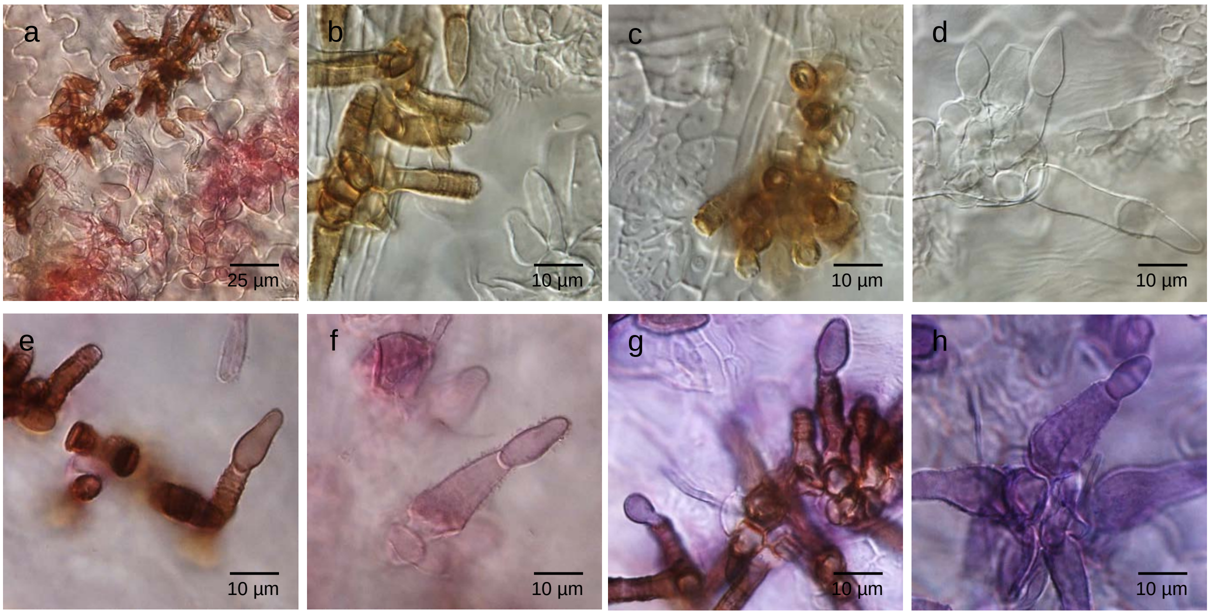

Figure S2. Staining of condiophores and conidia of *Venturia inaequalis* as influenced by the pigmentation of the wild type HS1 and the melanin-deficient mutant SW01, respectively. Mixture of both isolates sporulating on apple leaves 8 to 10 d p.i. (a-c; a stained with acid fuchsine); subcuticular hyphae, conidiophores and conidia of SW01 unstained (d), stained with acid fuchsine (f), stained with a mixture of acid fuchsine and aniline blue (h); conidiophores and conidia of HS1 stained with acid fuchsin (e), stained with a mixture of acid fuchsin and aniline blue (g).
